# Taurine reduces ER stress in C. elegans

**DOI:** 10.1186/1423-0127-17-S1-S26

**Published:** 2010-08-24

**Authors:** Hye Min Kim, Chang-Hee Do, Dong Hee Lee

**Affiliations:** 1 Department of Life Sciences, University of Seoul, Dongdaemun-Gu, Seoul, 130-743, Korea; 2 College of Agriculture and Science, Chungnam National University,Yuseong, Daejeon, 305-764, Korea

## Abstract

**Background:**

ER stress is a strong indicator of whether or not a cell is undergoing physiological stress. C. elegans is a practical system of characterizing the effect of ER stress at the *in vivo* or organismal level.

**Methods:**

This study characterized taurine’s anti-ER stress potential employing western blotting on ER stress markers and assays of motility, lifespan comparison, and fecundity measurement.

**Results:**

When treated with tunicamycin, C. elegans showed the typical ER stress symptoms. It showed a higher expression of hsp-70 and skn-1 than the non-treated control. Survivorship significantly decreased under tunicamycin treatment, and the offspring number also decreased. During the synchronized culture under ER stress conditions, the C. elegans showed early signs of aging especially between L3 and L4 within their life span, along with lowered motility. The worms, however, showed a positive response to the taurine treatment under ER stress conditions.

**Conclusions:**

When C. elegans were treated with taurine before or after the tunicamycin treatment, they showed a less severe level of ER stress, including an enhanced survivorship, increased motility, and augmented fecundity. Taken together, these results strongly indicate that taurine works positively to cope with ER stress from the organismal perspective.

## Background

Taurine is known to help cells recover from damage, and to prevent physiological stress by adjusting osmolarity. Under cellular stress conditions, taurine maintains the cellular homeostasis by achieving an osmotic balance within the cells; in detail, by controlling the functional gating of the ion channels involved in the intercellular ion trafficking [1]. No concrete mechanism, however, is available to explain the ability of taurine to counteract the harmful effects of physiological stress, such as endoplasmic reticulum (ER) stress.

ER stress symptoms serve as legitimate indicators of whether or not cells are undergoing physiological stress [2-5]. ER stress has been well characterized in terms of cellular response to various causative agents. The in vivo effect of ER stress is poorly understood, however, although a mouse model has been developed to monitor ER stress in vivo. There have been significant inconsistencies between in vitro and in vivo experiments on the ER stress response. This strongly implies that the organismal response represents the mixture of different cellular outcomes, and that the in vivo ER stress response may employ different elements along the pathway.

Despite the simple body scheme of C. elegans, it has been proven as sharing numerous vital biological pathways with mammals. It has become a valuable animal system in gaining an integrated understanding of organismal reactions to various forms of environmental and physiological stress [5-7]. Due to its short generation period, it enables researchers to study the effect of certain agents or conditions on aging and fecundity [8,9]. Under certain circumstances, C. elegans provides a practical system of studying both taurine’s effect on various ER stress responses at the organismal level and C. elegans’ anti-ER stress capability.

To characterize the potential anti-ERS mechanism of taurine, C. elegans was treated with tunicamycin, an ER stress inducer. Under the induced ER stress conditions, the effect of taurine was studied by monitoring the difference in the expression between ER stress marker protein (hsp-70) and the factor mediating adaptive responses to cellular stress (skn-1). The skn-1 is known to improve the ER stress conditions that negatively affected the lifespan, mobility, and fecundity of the C. elegans [10]. These three categories of stress physiological marker were also used to characterize whether taurine serves as an anti-ER stress mediator.

## Methods

C. elegans were normally grown at 25^o^C in the nematode growth medium (NGM) [11-13]. To induce ER stress conditions, worms were treated with tunicamycin at 10 μg/ml. To assay the effect of taurine, the worms were incubated with various extracellular taurine concentrations after they were treated for 12 h under ERS conditions. The ER stress conditions were verified by monitoring the ER stress marker expression. Equal homogenized samples were electrophoresed on a 10% SDS PAGE. Protein expression was quantified and standardized to the expression of actin protein. The values of the relative expression were obtained against the control treatments. The expression of skn-1 and hsp-70 was detected via western blotting using antibodies that were purchased from Santa Cruz Biotechnology (Santa Cruz, CA, USA). The western blotting was carried out according to the standard procedure, and the antibodies were diluted at 1:1,000.

The lifespan of the C. elegans was determined according to the method that Hyun *et al.* used [14]. After the worms were sacrificed by bleaching, 10 eggs were placed on NGM supplemented with OP50 at 25°C up to the young adult stage. Approximately 50 worms were placed on plates that contained 0-10 μg/ml of tunicamycin for 3 h. Then they were transferred to media that contained 10 and 100 μg/ml of taurine. They were monitored until they no longer responded to gentle stimulation with a platinum wire. For all the lifespan experiments, the assays were repeated twice.

To analyze the effect of taurine on the mobility of taurine, the distance of the movement of the worms that were treated in the presence of taurine and the distance of movement of the worms that were treated without taurine were compared. Initially, the worms were treated with 10 μg/ml of tunicamycin and transferred to taurine-containing media. Following the worms’ relocation to the taurine media, their total moving distance was determined from the track they had made.

The numbers of eggs that were laid were compared after the taurine treatment under the ER stress conditions. The worms were maintained on NGM plates covered with a lawn of OP-50 until they showed fresh moult. The adults were selected for uniformity and transferred to a fresh plate that contained 5 μg/ml of tunicamycin. Following their incubation for 6 h, they were transferred to taurine-containing media. Their fecundity was measured according to the combined number of their fertilized eggs and larvae for 3 days.

## Results and discussion

This study evaluated the anti-ER stress effect of taurine by determining if it is capable of reducing the stress caused by tunicamycin. The expression of hsp70, which was high after the tunicamycin treatment, considerably decreased under the taurine treatment in a dose-dependent manner. The skn-1 expression decreased when the worms were treated with taurine. In terms of the organismal markers, taurine showed an ER stress relieving effect by restoring the level of survivorship, fecundity, and motility of the worms.

### ER stress marker expression was reduced when worms were treated with taurine

**Figure 1:**
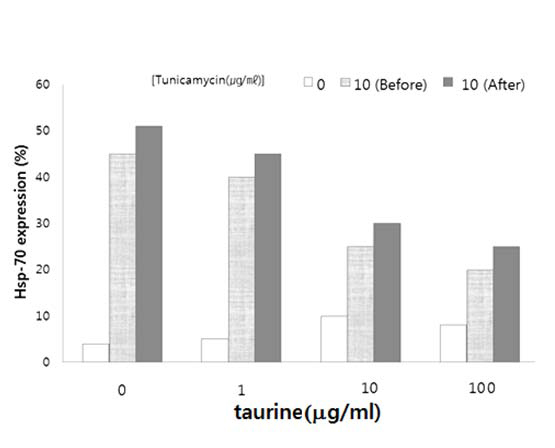
**Heat shock protein expression under taurine treatment.***C. elegans* were treated with taurine for 12 h after they had been cultured with tunicamycin. The hsp-70 expression was compared among four taurine concentrations. Protein expression was quantified and standardized to actin protein expression.

Based on the ER stress marker expression, taurine appears to have exerted an inhibitory effect on the progression of ER stress. When the cells were treated with taurine after their ER stress exposure, the level of hsp-70 significantly increased relative to the other ER stress markers. The significant upregulation of the ER stress markers suggests that taurine retards ER stress. The level of hsp70 expression showed typical dose-dependence, along with the amount of the tunicamycin that was added (Fig. 1). When the worms were incubated with various taurine concentrations, however, taurine downgraded the hsp-70 protein expression depending on the taurine concentration. The data imply that taurine lessens the intensity of ER stress caused by tunicamycin.

### The Increase of skn-1 is evident under taurine treatment

**Figure 2:**
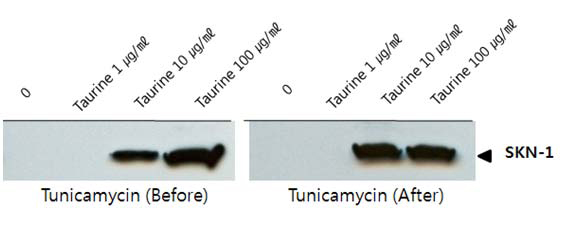
**Skn-1 expression** Worms were cultured with taurine either *before* or *after* treatment with tunicamycin. The expression of skn-1 protein expression was detected via western blotting.

The expression of skn-1 increased after the tunicamycin treatment, albeit differently from hsp70; but its expression significantly increased under the taurine treatment in a dose-dependent manner (Fig. 2). With regard to skn-1, however, the timing of the taurine treatment affected the expression of skn-1. When taurine was applied after tunicamycin, taurine had a more instantaneous effect on the level of the skn-1 expression (right panel) than when they were pre-treated with taurine (left panel). The results indicate that taurine is capable of lowering the level of ER stress by an increase in the level of skn-1, an anti-oxidative stress protein. This observation strongly implies that an existing ER stress response accelerates the expression of skn-1 by factors from the stress or by mechanisms to be further elucidated.

This oxidative stress response is very important as a cellular defense function and appears to be widely conserved during evolution. Oxidative stress induces skn-1 to accumulate in intestinal nuclei. Skn-1 is distantly related to the nrf (nuclear receptor factor) proteins that induce phase II detoxification gene transcription in mammals. The increase in skn-1 upon taurine treatment has a significant meaning, since ER stress could be diminished, as evidenced in Fig. 1, by the induction of detoxification gene expression, or by augmented expression of skn-1.

 Tunicamycin causes an increment in the reactive oxygen species (ROS), along with ER stress. Increased skn-1 expression may not help worms avoid the toxicity of tunicamycin-causing oxidative stress [15-18]. Should taurine’s efficacy as anti-ER stress agent be validated in subsequent experiments, this result would strongly imply that taurine may help lessen ER stress via the de novo anti-oxidative stress pathway.

The expression of hsp-70 decreased under taurine treatment, but that of skn-1 increased. The increase in the skn-1 expression with taurine treatment is confusing, since its higher expression may refer to an increasing level of ER stress under taurine treatment [19-21]. Unlike hsp-70, skn-1 may play a more functional role in lessening the severity of ER stress than other ER stress markers.

### Taurine prolongs the lifespan of ER stressed worms

**Figure 3:**
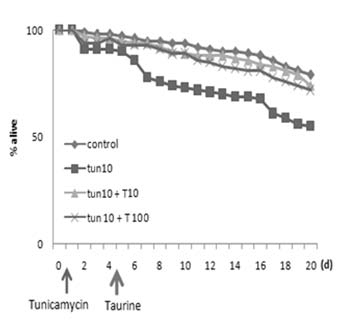
**Life span assay** Worms were grown on taurine-containing media (1 or 10 mg/ml) following the treatment with tunicamycin (10 μg/ml). Survivorship was recorded daily following taurine treatment of the worms which were subjected to ER stress. The percentage of live worms greatly increased in a dose dependent fashion.

The worms’ lifespan was negatively affected by their treatment with tunicamycin. When the treatment began one day after the initiation of the culture, the worms responded sensitively to tunicamycin, and many of them died (Fig. 3). After they were treated with tunicamycin alone, half of them died within 20 days after the start of the treatment. The taurine treatment, however, greatly improved the worms’ survival rate. No difference was seen between the tunicamycin and the tunicamycin-free treatment groups. Depending on the concentration of the taurine treatment, however, the worms recovered their lifespan up to the level where no tunicamycin was applied.

### Taurine helps restore the worm’s fecundity

**Figure 4:**
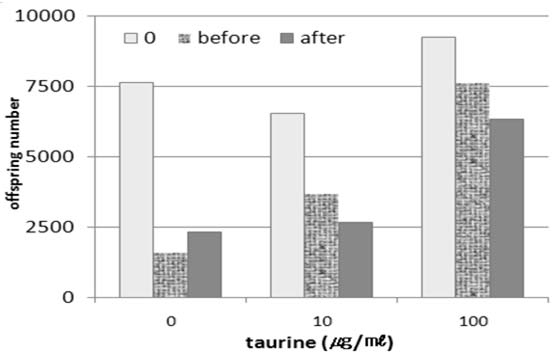
**Fecundity assay** Under taurine treatment, tunicamycin-induced stress appeared to decrease in terms of fecundity. Each value represents the mean of three experiments. Taurine appeared more effective when it is applied *before* the addition of tunicamycin than the “after” treatment (10 μg/ml).

Taurine’s effect on fecundity was measured following its application to tunicamycin-treated worms. After the tunicamycin treatment, the worms laid much fewer eggs. When the worms were cultured on taurine media, however, their egg-laying ability normalized (Fig. 4). This increased level of fecundity could be attributed to taurine’s reduction of the level of ER stress [22-24].

### Taurine helped C. elegans recover from tunicamycin-reduced motility

**Figure 5:**
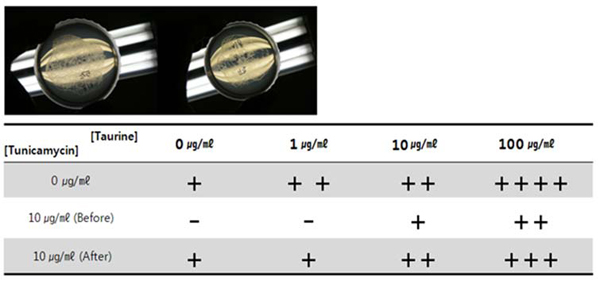
**Motility comparison** The extent of worms’ movement was visually compared among various taurine treatments. The upper panel shows the different degrees of mobility between taurine (*left*) and taurine-free treatment (*right*). Although tunicamycin retarded worm’s movement, its negative effect was compensated by taurine in the media (*lower panel or table*).

When the C. elegans were treated with tunicamycin, their mobility more significantly decreased than with the non-tunicamycin control. They substantially recovered their movement ability, however, within one hour after the transfer. The values of their total moving distance for different concentrations of media-applied taurine were compared. Fig. 5 shows the different effects of the taurine and the taurine-free treatment with different doses. This result strongly implies that taurine helped the worms recover from the negative effect of ER stress on their muscular activity [25-28].

## Conclusions

Upon treatment with tunicamycin, C. elegans showed typical ER stress symptoms, such as elevated expression of heat shock proteins (Hsp-70). Skn-1 expression increased and induced an anti-oxidative stress pathway. The tunicamycin treatment also caused many physiological stress symptoms: decreased survivorship, retarded movement, low fecundity, and early aging. When the worms were treated with taurine, however, they showed positive responses against ER stress conditions. They showed less ER stress, longer survivorship, and improved mobility and fecundity. These results strongly indicate that taurine fights ER stress from the in vivo physiological perspective.

## List of abbreviations used

ER: endoplasmic reticulum; nrf: nuclear receptor factor; hsp: heat shock protein; NGM: nematode growth media

## Competing interests

The authors declare that they have no competing interests.

## Authors' contributions

HMK carried out the maintenance of C. elegans and participated in the taurine treatment and data collection. CHD participated in the design of the study and performed the statistical analysis. DHL participated in obtaining worm strains and coordination of the experiments and helped to draft the manuscript. All authors read and approved the final manuscript.
